# Identification and physiological activity of (methoxymethyl)triphenylphosphonium chloride as a new phytotoxin isolated from *Rhizoctonia solani* AG-3 TB

**DOI:** 10.3389/fpls.2023.1264567

**Published:** 2023-11-17

**Authors:** Xinchun Li, Huihui Hou, Bin Li, Shiping Guo, Lianqiang Jiang, Chuantao Xu, Yunbo Xie, Mengnan An, Chong Zhang, Yuanhua Wu

**Affiliations:** ^1^ Liaoning Key Laboratory of Plant Pathology, College of Plant Protection, Shenyang Agricultural University, Shenyang, China; ^2^ School of Economics and Management, Liaoning University of Technology, Jinzhou, China; ^3^ Quality Inspection Department, Sichuan Province Tobacco Company, Chengdu, China; ^4^ Quality Inspection Department, Liangshan Branch of Sichuan Province Tobacco Company, Xichang, China; ^5^ Quality Inspection Department, Luzhou Branch of Sichuan Province Tobacco Company, Luzhou, China

**Keywords:** (methoxymethyl)triphenylphosphonium chloride (MMC), *Rhizoctonia solani* AG-3 TB, phytotoxin, pathogenic mechanism, toxin compound

## Abstract

*Rhizoctonia solani* as a cosmopolitan fungus is the causative agent of many crop diseases and leads to significant economic losses in crop production. To explore the toxin structure and physiological activity of *R. solani* AG-3 TB, high-performance liquid chromatography (HPLC), infrared absorption spectrum (IR), and nuclear magnetic resonance spectrum (NMR) were required. Here, the compound (methoxymethyl)triphenylphosphonium chloride (MMC) with the molecular formula C_20_H_20_ClOP was purified and identified from *R. solani* AG-3 TB. The pure compound MMC treated at 20 μg/mL, 50 μg/mL, and 100 μg/mL can cause obvious necrosis on leaves, increase active oxygen species (AOS), decrease chlorophyll content, and damage cellular structure. The results enrich the understanding of toxin compounds for *R. solani* and provide valuable insights into the toxicology of *R. solani* AG-3 TB.

## 1 Introduction

*Rhizoctonia solani* (*R. solani*) belongs to basidiomycete species with a large host range and different anastomosis groups. In suitable environmental conditions, *R. solani* can cause serious diseases on the stems, leaves, and roots of many plants. It is divided into 14 subgroups based on its special genetic and biological characteristics, including AG-1 to AG-13 and a bridging isolate AG-BI (Taheri and Tarighi, 2011), among which *R. solani* AG-1 IA and AG-3 PT subgroups cause black scurf and stem canker on the stem and roots of rice and potato, respectively (Salamone and Okubara, 2020), while yellow-brown lesions with concentric rings can be induced on leaves of tobacco by *R. solani* AG-3 TB subgroups, which was known as tobacco target spot disease (Elliott et al., 2008; Wu et al., 2012). Tobacco target spot disease was first recorded in the United States, and it occurred subsequently in South Africa, Italy, and Canada (Lucas, 1975; Meyer et al., 1990; Reeleder, 1996; Kuninaga et al., 2000). In 2012, dark-brown lesions were discovered in a tobacco field in Kuandian and Fengcheng Counties, Dandong City of Liaoning in northeast China, which was identified to be tobacco target spot disease (*R. solani* AG-3 TB) by physiological and molecular methods (Wu et al., 2012). At present, this disease has been discovered in Guizhou, Yunnan, and Sichuan provinces, and the area of the tobacco field has gradually expanded (Xu et al., 2018; Xu et al., 2021).

*R. solani* as a common pathogen in nature can secrete pathogenic factors, such as toxins, to inhibit the growth of plants, destroy normal plant metabolism, and reduce crop yield (Doehlemann et al., 2017). In the middle of the 20th century, succinic acid, phenylacetic acid (PAA), and furan acid were first isolated and identified to be the main toxin compounds in *R. solani* AG-1 IA (Aoki et al., 1963). Then, *m*-hydroxy and *o*-hydroxy-phenylacetic acid caused wilting of plants and other toxic effects and were the two potentially toxic compounds separated from *R. solani* AG-4 (Mandava et al., 1980). 3-methylthiopropionic acid (3-MTPA) isolated from *R. solani* AG-3 PT can destroy the cell membrane and cause cytoplasmic breakage in potatoes (Kankam et al., 2016). In our previous study, we revealed that 3-methoxyphenylacetic acid (3-MOPAA) is an important toxin compound during *R. solani* AG-3 TB infection, and its pure compound at different concentrations of 1 mg/mL, 2 mg/mL, and 4 mg/mL can cause necrotic lesion on leaves of tobacco (Var.: NC89) (Li et al., 2023).

Due to the special genetic and physiological characteristics of *R. solani*, the pathogenic mechanism such as toxin compounds and toxin synthesis process remains unclear. Therefore, the exploration of pathogenic toxin compounds and synthetic pathways of toxins has become an important direction to control the disease’s spread. Currently, the compound including phenylacetic acid (PAA), *m*-hydroxy, and *o*-hydroxy-phenylacetic acids was identified in different anastomosis groups of *R. solani*. However, other potential pathogenic compounds are still unclear in *R. solani* AG-3 TB besides 3-methoxyphenylacetic acid (3-MOPAA) identified in our previous study. Here, we clarified another pathogenic novel compound, (methoxymethyl) triphenylphosphonium chloride (MMC), produced by *R. solani* AG-3 TB based on high-performance liquid chromatography (HPLC), infrared absorption spectroscopy (IR), and nuclear magnetic resonance spectroscopy (NMR). The pure compound MMC at different concentrations of 5 μg/mL, 10 μg/mL, 20 μg/mL, 50 μg/mL, and 100 μg/mL could induce necrotic lesions on leaves and damage the normal physiological activity of plants. This result enriches the research on the toxin of *R. solani* and provides a new direction for the in-depth exploration of *R. solani*.

## 2 Materials and methods

### 2.1 Isolation, purification, and identification of the toxin structure

In our previous study, the toxin was extracted from *R. solani* AG-3 TB YC-9 strain with activated carbon adsorption extraction, and the purified toxin compound was tested by Thin-Layer Chromatography (TLC) (Li et al., 2023). Then the toxin sample was prepared by the KBr compression method. KBr and 1.8 mg of toxin sample were ground into powder and made into a sheet. The infrared absorption peak was determined by the Nicolet 6700 Fourier transform spectrometer (Thermo Fisher Company, USA).

The mass charge ratio (m/z) of the toxin sample was obtained by the combined quadrupole Orbitrap mass spectrometer (Q Exactive™). Distilled water was used as a mobile phase solvent, and ESI (−) ionization mode was adopted. The toxin sample was measured by the German Elemental Verio MICRO cube element analyzer. The sample was decomposed, quantified, and transformed; finally, the percentage content of C, H, O, and N was detected. The toxin structure was analyzed by ^1^H-NMR and ^13^C-NMR spectra.

### 2.2 Pathogenicity of MMC on plants

The MMC with 5 μg/mL, 10 μg/mL, 20 μg/mL, 50 μg/mL, and 100 μg/mL were used for inoculation on leaves of *Nicotiana tabacum* (Var.: NC89), and sterile water was used as control. The leaves inoculated with MMC were cultured at 28° for 3 d and the lesion diameters were measured. Each treatment was tested three times. A total of 10 tobacco experiments were performed, and each leaf was inoculated at four acupuncture points including two sterile water acupuncture points as control and two MMC acupuncture points as the treatment group.

### 2.3 Determination of active oxygen species

The MMC solution of 5 μg/mL, 10 μg/mL, 20 μg/mL, 50 μg/mL, and 100 μg/mL was injected into the leaves according to the infiltrating method (Arnon, 1949). The treated leaves were cultured in a dish soaked with three layers of sterile gauze at 28°, and the samples were collected at an interval of 8 h.

The leaf of 0.05g was dissolved with 1 mL phosphate buffer solution of 56 mmol/L and fully pestled. The extraction was centrifuged at 4° and 12000 rpm for 8 min, and 1mL of 1 mol/L light amine hydrochloride (pH 7.8) was dissolved with 0.5 mL supernatant and incubated for 1 h at 25°. Then, p-aminobenzene sulfonic acid of 1 mL with 17 mmol/L and 1 mL of 7 mmol/L 1-naphthylamine were added and cultured at 25° for 25 min. The OD_530_ number of this mixture was measured. The amount of different substances in the standard solution (n) was measured as NO^2−^ standard curve, the absorbance was measured as the ordinate, and the amount of NO^2−^ was used as abscissa. According to the chemical reaction equation (μmol·g^−1^·min^−1^), the production rate of superoxide ions (O^2−^) was calculated.

Chemical reaction equation (μmol·g^−1^·min^−1^) = (C×V×1000)/(Vs×t×W)

C – number of superoxide anion in solution, μmol;
V – Total volume of sample, mL;
Vs – Measure sample volume, mL;
t – The reaction time between the sample and hydrochloride, min;
W – The sample weight, g.

The H_2_O_2_ production in leaves was conducted according to the constant volume colorimetry method (Patterson et al., 1984). Using 2 mL of precooled acetone, 0.05 g of leaf tissue was attrited evenly. The exaction was centrifuged at 4°, 12000 rpm, for 15 min. As the sample extraction solution, 1mL of supernatant was absorbed, and 1 mL of 5% titanium sulfate and 1 mL of concentrated ammonia water were added into the extraction solution. The supernatant was removed, and the precipitate was washed with acetone (three to five times) and dissolved by 4 mL H_2_SO_4_ (2 mol/L). The solution’s constant volume colorimetry (415 nm) was measured.

Hydrogen peroxide content in leaf tissue (μmol/g Fw) = (C×Vo)/(Fw×Vt)

C – the sample on the standard curve of H_2_O_2_ concentration (μmol/L)
Vo – volume of sample extract (mL)
Vt – volume of supernatant used for determination (mL)
FW – weight of leaf tissue (g)

### 2.4 The chlorophyll content in *N. tabacum* leaves treated by MMC

The leaves were put into 7 mL of pure toxin solution with different concentrations of 5 μg/mL, 10 μg/mL, 20 μg/mL, 50 μg/mL, and 100 μg/mL and cultured at 28° in light conditions for 3 days. Then, 0.2 g of leaf tissue was cut into pieces, and the mixture of acetone [ethanol (V/V=1:1) (10 mL)] was infiltrated and incubated for 24 h in dark conditions. When the color of leaves was observed from green to white, the OD_645_ and OD_663_ were measured. The leaves in sterile water were used as a control, and each treatment was repeated three times.

Chlorophyll content in leaf tissue (Co) = [(20.29×OD_645 _+ 8.05×OD_663_)×V]/(1000×W)
V – volume of chlorophyll acetone extract (mL)
W – Fresh weight of leaf tissue (g)

### 2.5 The cell structure of *N. tabacum* leaves treated with MMC

Twenty microliter MMC at 50 μg/mL was injected into tobacco leaves (Var.: NC89). The injected leaves were incubated at 25° in dark conditions. Then leaves were washed with distilled water (two to three times), cut into 2 mm × 1 mm segments, and dissolved with 2.5% glutaraldehyde solution at 4° for 24 h. For 15 min dehydration, 70% and 80% ethanol solutions and 85%, 95%, and 100% acetone were used respectively, and the samples were embedded with EPon812 resin. The tissue was stained with 2% uranyl acetate lead citrate, and the ultrastructural changes of tobacco mesophyll cells were observed by transmission electron microscopy (TEM) (HT7700, HITACHI, Japan).

## 3 Results

### 3.1 Isolation and purification of the toxin compound of *R. solani* AG-3 TB

**Figure 1:**
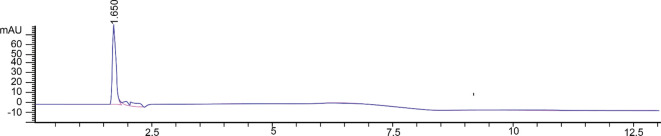
Toxin compound I isolated from *R. solani* AG-3 TB analyzed by HPLC.

According to our previous study, the radicle elongation and lesion diameter of tobacco was inhibited by the crude toxin extraction from *R. solani* AG-3 TB, and the toxin extraction was analyzed by thin-layer chromatography and HPLC (Li et al., 2023). Then the compound I was collected by preparative HPLC and the single peak could be detected (**Figure 1**).

### 3.2 Structure identification of the toxin compound

**Figure 2:**
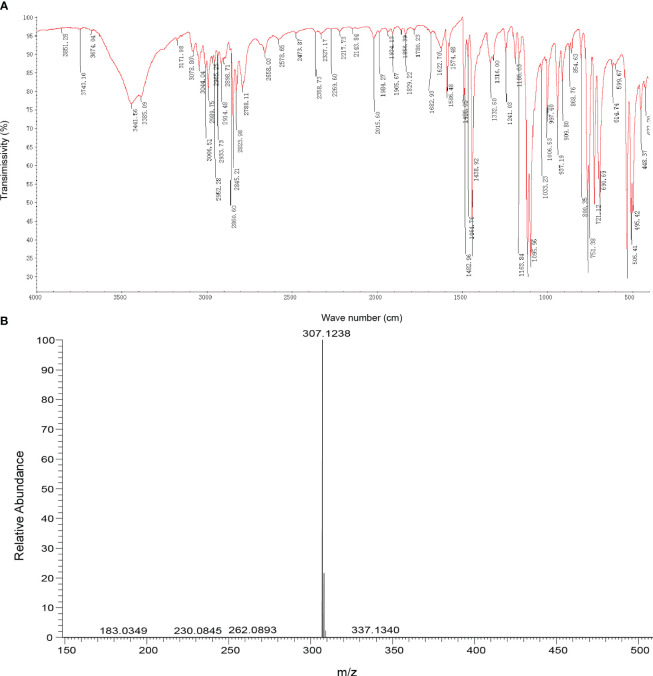
Identification of toxin compound by IR and mass spectrometry. **(A)** Toxin compound analysis by IR spectrum. **(B)** Toxin compound analysis by mass spectrometry.

Infrared absorption spectroscopy (IR) was used to identify the toxin compound structure. According to the IR spectrum, -C-H telescopic vibration and C-H bending vibration were observed at 2990 cm^−1^, 2952 cm^−1^, 2845cm^−1^,1465 cm^−1^, and 1439 cm^−1^, which indicated that -CH_2_- and -OCH_3_ existed in the structure. The result indicated that the benzene ring structure, especially monosubstituted benzene, was identified in this structure on the basis of =C-H telescopic vibration, benzene ring skeleton C=C, and =C-H out of plane bending vibration determined at 3044 cm^−1^, 1622 cm^−1^, 1586 cm^−1^, 1483 cm^−1^, 752 cm^−1^, 721 cm^−1^, and 691 cm^−1^. At ∼1120 cm^−1^, ^+^P-C telescopic vibration existed, while at 1096 cm^−1^, C-O-C telescopic vibration was observed; therefore, the ^+^P-Ar and dialkyl ether (ROR’) were included in this structure. According to the result of IR, monosubstituted benzene, ROR’, ^+^P-Ar, -CH_2_-, and -OCH_3_ were found in the toxin component (**Table 1**; **Figure 2A**).

**Table 1:** Element composition of toxin compound by IR.

Wave number of absorption peak (cm^−1^)	Vibration type	Group	Absorption peak intensity
3400	=C-H Telescopic vibration	=C-H	m
2990, 2952, 2845	-C-H Telescopic vibration	Saturated -C-H	s, m, s
1622, 1586, 1483	Benzene ring skeleton C=C	Benzene ring	m, s, s
1465	C-H Bending vibration	-CH2-	s
1439	C-H Bending vibration	-OCH3	s
~1120	C-O-C Telescopic vibration	Dialkyl ether ROR’	s
1096	^+^P-C Telescopic vibration	^+^P-Ar	s
752, 721, 691	=C-H Out of plane bending vibration	Monosubstituted benzene	s, s, s
1234	O-H Bending vibration	Carboxylic acid (-COOH)	s
881, 793, 704	=C-H Out of plane bending vibration	1,3- disubstituted benzene	s, s, s

According to mass spectrometry, the mass-to-charge ratio of the M^+^ peak of the toxin sample was 307.1 (**Figure 2B**), and the molecular weight of the phosphate cation in the compound was 307. Based on the percentage content of elements C, H, and O, the number ratio of elements C, H, and O in the compound was calculated to be 20:20:1 (**Table S1**). Combined with the result of IR, the molecular formula of the toxin compound was consistent with C_20_H_20_ClOP.

**Figure 3:**
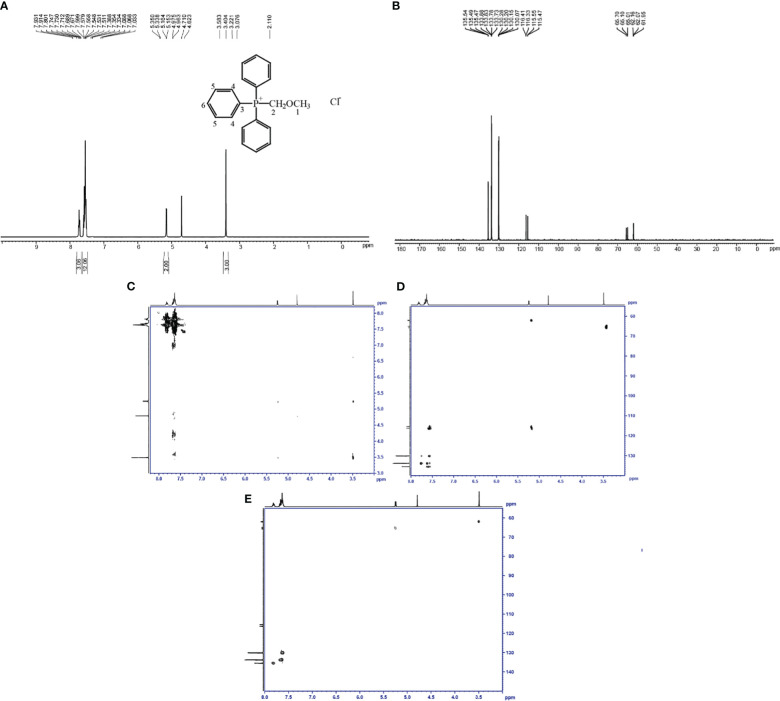
Identification of the toxin compound structure isolated from *R. solani* AG-3 TB. **(A)** Toxin structure analyzed by ^1^H-NMR. **(B)** Toxin structure analyzed by ^13^C-NMR. **(C)** COSY NMR spectrum of toxin compound. **(D)** HMBC NMR spectrum of toxin compound. **(E)** HSQC NMR spectrum of toxin compound.

The structure of the toxin compound was deduced by nuclear magnetic resonance (NMR), and ^1^H-NMR results (**Figure 3A**; **Table S2**) revealed that four groups of peaks were identified and their integral ratio (from low field to high field) was 3:12:2:3, and the total number of protons was 20. According to the results of chemical shift values and COSY and HMBC experiments (**Figures 3C, D**), the structure of toxin compound had a spin system composed of 15 aromatic protons on three monosubstituted benzenes (δ 7.81 (3H, m), 7.65 (6H, m), and 7.62 (6H, m)), which were 3H_6_,6H_4_, and 6H_5_, respectively. On the base of δ5.24 (2H, d) and 3.49 (3H, s) values, there were two groups of isolated protons, which were H_2_ and H_1_, among which H_2_ was split into two peaks by the coupling splitting of P ion.

The results of the carbon spectrum, displacement table, and HSQC two-dimensional spectrum (**Table S3**; **Figures 3B, E**) indicated that two saturated Cs (δ 65.4(d) and 62.0(d)) were observed in the structure, which were C_2_ and C_1_, and they were split into two peaks by the coupling splitting of P ion. There were three kinds of 15 aromatic tertiary C according to the value of δ130.1(d), 133.8(d), and 135.5(d), which were 6C_5_, 6C_4_, and 3C_6_, and they were isolated by the coupling splitting of P ion. One kind of three unsaturated seasons C was detected at δ 115.9(d); they were 3C_3_, which were split into two peaks by the coupling splitting of P ion. According to the results of NMR, the structure of the toxin compound was consistent with (methoxymethyl)triphenylphosphonium chloride (MMC) (C_20_H_20_ClOP) (**Figure 3A**).

### 3.3 Pathogenicity of pure compound MMC on *N. tabacum*

**Figure 4:**
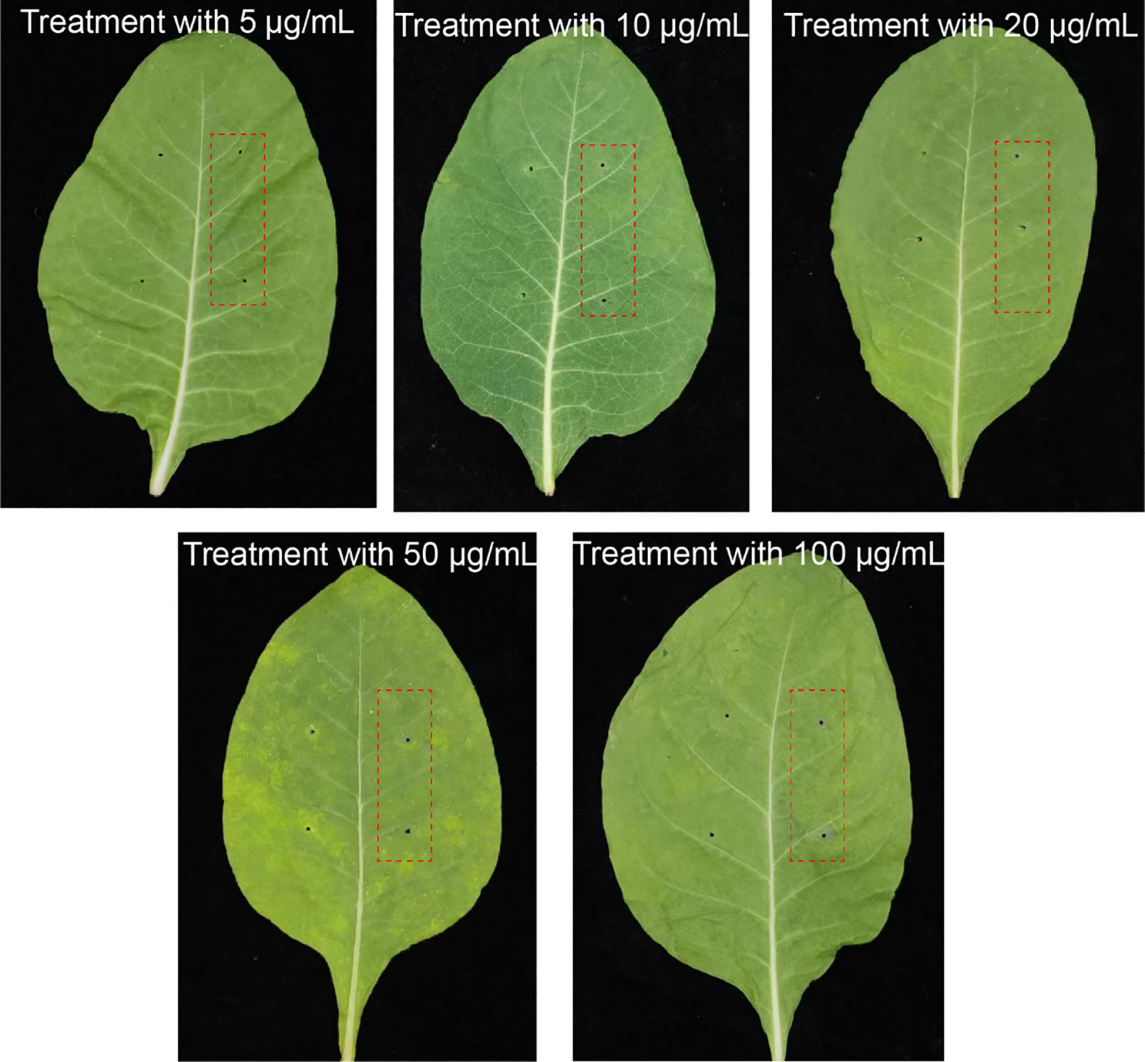
Symptoms of *N. tabacum* treatment with different pure MMC solutions. The two needling points on the left were treated with sterile water used as control, while the red frame indicates treatment groups by pure compound MMC.

The pure compound MMC at the concentrations of 5 μg/mL, 10 μg/mL, 20 μg/mL, 50 μg/mL, and 100 μg/mL was treated on leaves, and the lesion diameter was measured to clarify its pathogenicity and virulence. The results indicated that 100 μg/mL of MMC can cause obvious necrosis around the inoculation point on leaves. The lesion diameters measured were 0.175 ± 0.120 cm, 0.263 ± 0.057 cm, 0.366 ± 0.072 cm, 0.425 ± 0.276 cm, and 0.506 ± 0.185 cm, respectively, after treatment with 5 μg/mL, 10 μg/mL, 20 μg/mL, 50 μg/mL, and 100 μg/mL of MMC (**Figure 4**; **Table 2**).

**Table 2:** Lesion diameters caused by MMC at different concentrations.

Compound Treatment	5 μg/mL	10 μg/mL	20 μg/mL	50 μg/mL	100 μg/mL	CK
Lesion diameter (cm)	0.175 ± 0.120	0.263 ± 0.057	0.366 ± 0.072	0.425 ± 0.276	0.506 ± 0.185	0.00

### 3.4 Effects of pure compound MMC on the production of active oxygen species

**Figure 5:**
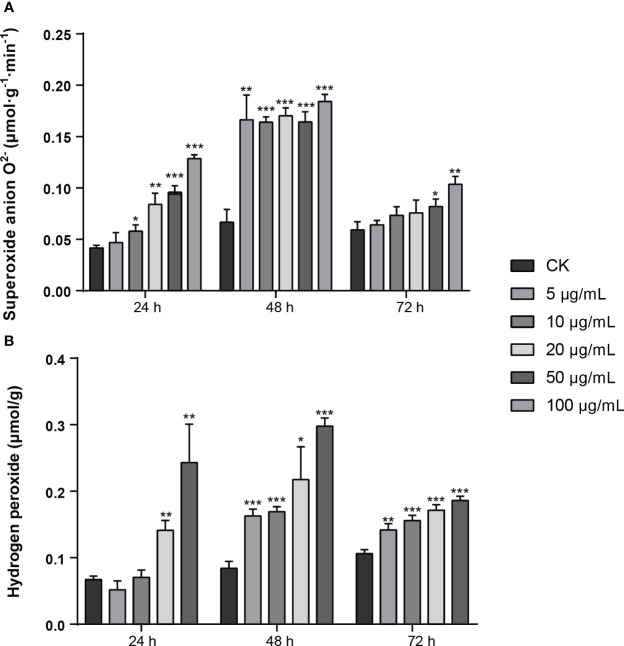
Production of active oxygen species (AOS) of MMC treatment on *N. tabacum*. **(A)** Production rate of O^2−^ after treatment with pure compound MMC. **(B)** Production of H_2_O_2_ after inoculation with pure compound MMC. A two-tailed t-test was used to determine statistical significance (*: *p*: 0.05, **: *p*: 0.01 ***: *p*: 0.001).

In order to clarify the effect of MMC (5 μg/mL, 10 μg/mL, 20 μg/mL, 50 μg/mL, and 100 μg/mL) on the production of active oxygen species (AOS) on leaves, the production rate of superoxide anion O^2−^ and content of hydrogen peroxide (H_2_O_2_) were determined (**Figure 5A**). The results showed that the production rate of O^2−^ in leaf tissue was increased at 24 h and 48 h and decreased at 72 h after treatment with MMC. In addition, the production rate of O^2−^ declined at 72 h, of which the number of rates were 0.082 μmol/g.min and 0.104 μmol/g.min treated by 50 μg/mL and 100 μg/mL of MMC. The production rate of O^2−^ treated with different concentrations revealed that the production rate of O^2−^ treatment by 20 μg/mL, 50 μg/mL, and 100 μg/mL of MMC were 2.01, 2.25, and 3.09 folds higher compared with the control at 24 h.

The content of hydrogen peroxide (H_2_O_2_) in leaves indicated that H_2_O_2_ content treated by MMC at 48 h was higher than other treatment groups, among which H_2_O_2_ content was 0.298 μmol/g treated by 50 μg/mL of MMC, of which comparison with the control was 3.55 folds higher. In addition, H_2_O_2_ content in leaves treated for 72 h was lower than those leaves treated for 24 h or 48 h, but H_2_O_2_ content in leaves during this period was still significantly higher than in the control group. The H_2_O_2_ content produced in leaves significantly changed after treatment with MMC, of which the H_2_O_2_ contents in leaves were 0.163 μmol/g, 0.169 μmol/g, 0.217 μmol/g, and 0.298 μmol/g, respectively, treated with 5 μg/mL, 10 μg/mL, 20 μg/mL, and 50 μg/mL of MMC inoculation at 48 h (**Figure 5B**).

Therefore, AOS content in leaves can be affected by the pure compound MMC; especially, the higher concentration of MMC can have a serious impact and change the content of H_2_O_2_ and O^2−^ in leaves.

### 3.5 Effects of pure compound MMC on chlorophyll content

Chlorophyll is the main component for capturing light in photosynthesis. In the study, the chlorophyll content in leaves treated with different concentrations of MMC was decreased significantly (**Table 3**). The decline ratio of chlorophyll treated by 5~20 μg/mL of MMC was not obvious, but the decline ratio of chlorophyll content treated by 50 μg/mL and 100 μg/mL of MMC were 42.35% and 56.13%, respectively. Therefore, the result revealed that chlorophyll content can be damaged by pure compound MMC identified from *R. solani* AG-3 TB, and the higher concentration of MMC when produced causes large damage to the chlorophyll content.

**Table 3:** Chlorophyll content in *N. tabacum* leaves treated by MMC.

Concentration (μg/mL)	Decline ratio of chlorophyll content (%)	Difference significance	
		5 (%)	1 (%)
CK	0	e	E
5	2.59	e	E
10	12.74	d	D
20	21.98	c	C
50	42.35	b	B
100	56.13	a	A

### 3.6 Effects of pure compound MMC on cell structure

**Figure 6:**
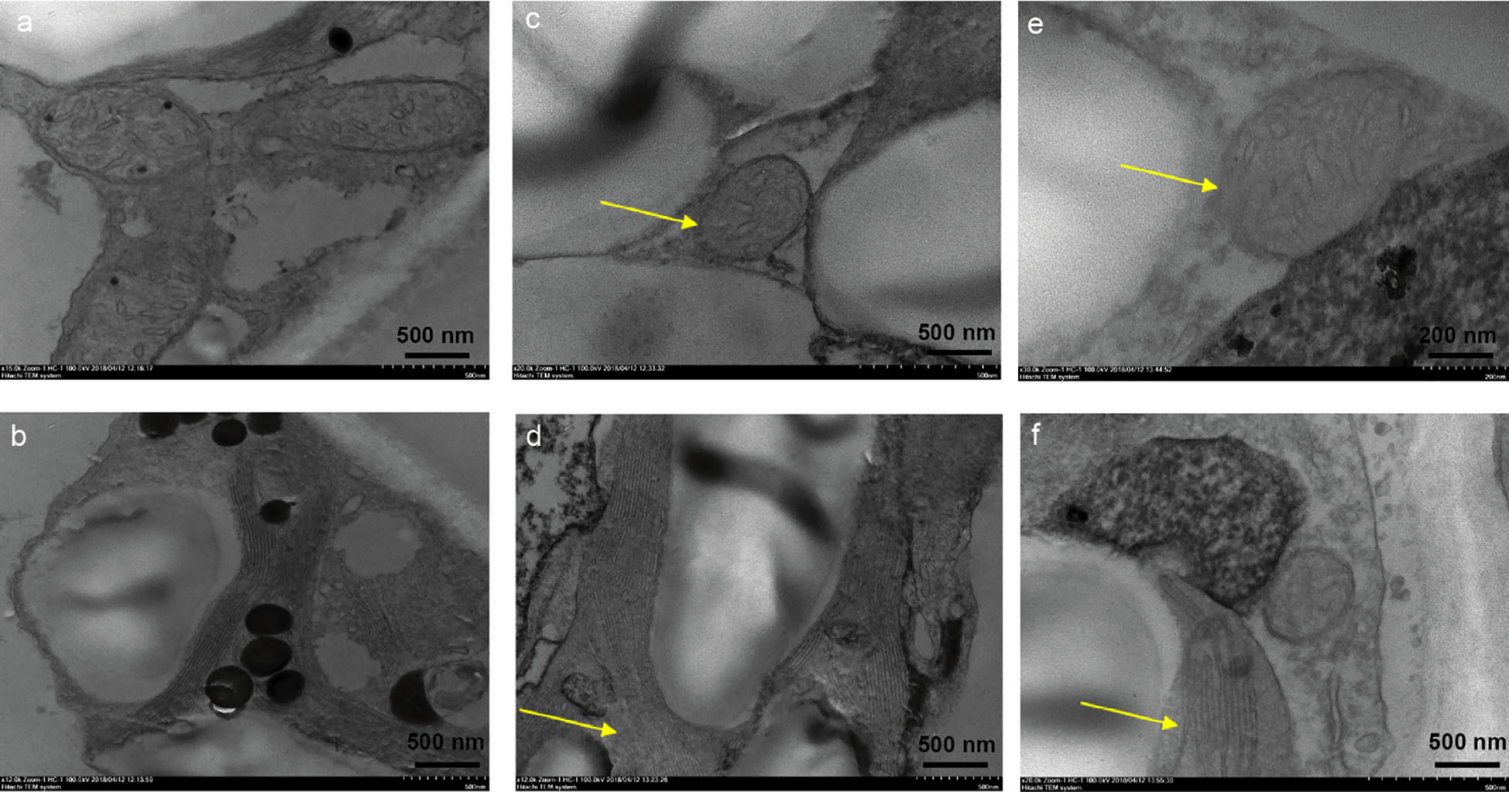
Effects of pure MMC (50 μg/mL) on *N. tabacum*. **(A)** The structure of mitochondrion treated by sterile water. **(B)** The structure of chloroplast treated by sterile water. **(C)** Mitochondrial cell thickening treated by MMC at 12 h. **(D)** Chloroplast lamella rupture treated by MMC at 12 h. **(E)** The structure of mitochondrion almost disappearing after treatment with MMC at 48 h. **(F)** Chloroplast lamella almost disappearing after treatment with MMC at 48 h.

In order to clarify the toxicity of phytotoxin of *R. solani* AG-3 TB to the mesophyll cell structure of leaves, the structure of chloroplast and mitochondria in leaves was observed after treatment with 50 μg/mL of MMC. The result indicated that compared with control results of leaves treated with sterile water (**Figures 6A, B**), the structure of the chloroplast and mitochondrial cell began to split in those treated with 50 μg/mL of MMC at 12 h (**Figures 6C, D**). Moreover, the cytoplasmic wall was seriously separated, and the structure of the chloroplast membrane and lamellar completely disappeared in leaves treated with MMC for 48 h (**Figures 6E, F**).

## 4 Discussion

Due to the special multi‐nuclear and multi‐subgroup genetic characteristics and differentiation background of *R. solani*, it is relatively difficult to explore pathogenicity in depth. However, the pathogenic factors including toxin compounds, effectors, and enzyme genes were confirmed during *R. solani* AGs infection. The toxin compounds of carboxylic acid in *R. solani* isolated and identified from AGs, such as phenylacetic acid (PAA), *o*‐hydroxy‐phenylacetic acid (*o*‐OH‐PAA), *m*‐hydroxy‐phenylacetic acid (*m*‐OH‐PAA), and 3‐methylthi-opropionic acid (3‐MTPA), were confirmed to be the main pathogenic toxin compound (Aoki et al., 1963; Adachi and Inagaki, 1988; Bartz et al., 2013; Kankam et al., 2016). Moreover, 3-methoxyphenylacetic acid (3-MOPAA) produced by the AG-3 TB subgroup can cause the chlorosis of leaves at 24 hpi, and expression levels of crucial enzyme genes in the PAA synthesis pathway increased dramatically (Li et al., 2022; Li et al., 2023). In this study, MMC was identified to be an important toxin compound secreted by AG-3 TB using HPLC, IR, and NMR. At present, there is no relevant report on MMC as secondary metabolites produced by microorganisms; however, it is interesting that this compound plays a key role in the Wittig reaction as a Wittig reagent, which is a valuable method for synthesis for aldehydes and ketones transforming to olefins. During the Wittig reaction, the aldehyde condensed with MMC, and the carbon-carbon single bond was transformed to the olefins with a carbon-carbon double bond, such as in the reaction of 7-acylamino-6,7-dideoxy-galactohepto-pyranoses; intermediate aldehyde was condensed with the Wittig reagent (methoxymethyl)triphenylphosphonium chloride, and a homologous heptodialdo-1,5-pyranose was observed after hydrolysis (Wünsch, 2003). In addition, the Wittig reaction also plays an important role in insect pheromone, in which in the synthesis of the production for rice stem borer pheromone, MMC was used for the Wittig reaction to obtain the intermediate product of 13 cis-1-methoxyoctadec-1,13-diene (Schlosser, 1970; Schlosser et al., 1985; Xiao et al., 2019). Therefore, we supposed that MMC in *R. solani* AG-3 TB may be an important reaction intermediate for producing some kind of double-bond alkenes, such as the synthesis of insect pheromones.

Active oxygen species (AOS) play several key roles in disease resistance, including the hypersensitive response in the plant–pathogen incompatible interactions, limiting the entry and transmission of pathogens by cell wall reinforcements or directly killing pathogens, and activating host defense signaling pathways (Levine et al., 1994; Chamnongpol et al., 1998; Dat et al., 2000). The different H_2_O_2_ kinetics in AOS can activate defensive genes, induce programmed cell death, and promote the production of salicylic acid (SA). In the process of interaction between plants and pathogens, the toxin with low concentration usually dramatically damages plants and even harms human health (Schafer, 1994; Nieto et al., 2018). However, some reported that fungal secondary metabolites have a close relation to the oxidative stress of plants, among which H_2_O_2_ plays a crucial role in regulating the production of toxic factors. For instance, the specific *Aspergillus flavus* strains grew well in the medium containing 0 to 50 mM H_2_O_2,_ and increasing H_2_O_2_ concentrations in the media resulted in elevated aflatoxin production in toxigenic isolates (Fountain et al., 2015). Moreover, the content of moniliformin (MON) and fumonisins (FBs) in corn can be effectively controlled after H_2_O_2_ treatment, which highlighted the direct impact of H_2_O_2_ on the stability of mycotoxins (Ferrigo et al., 2021). In this study, the content of AOS (H_2_O_2_ and O^2−^) increased after treatment by MMC identified from *R. solani* at increased concentrations. This result revealed that MMC can induce H_2_O_2_ production to stimulate plant defense and thereafter reduce the damage of the toxin to mesophyll tissue. At present, it is uncertain that the production of MMC identified from *R. solani* AG-3 TB has a direct relationship with AOS production in plants, but this compound may be a toxic compound that damages tissues.

Photosynthesis is critical for the synthesis of organic substances in plants. Solar energy and carbon dioxide can be absorbed by chlorophyll, which positively correlates with photosynthesis in plants (Suzuki et al., 1997; Eggink et al., 2001; Melis, 2009). Most toxic compounds can cause yellowing of leaves and reduce the chlorophyll content of plant leaves, such as higher MTPA concentrations decreased chlorophyll content of potato based on the data of the luminous energy, and electron transfer efficiency (Kankam et al., 2016). In addition, the cell structure is a complex environment, which can ensure the self-regulation and order of progress of life. The cell membrane and cytoplasm fracture corroborating can be caused by high levels of toxin concentration. Particularly, the mycotoxin of many necrotrophic pathogenic fungi causes cell death to facilitate the absorption of nutrients (Friesen et al., 2008). Based on the result of the study, pure MMC identified in *R. solani* AG-3 TB may be a key contributor to producing disease spots, and it was also an important toxin compound for the spread of leaf chlorosis.

Bioactive molecules produced by specific fungi are known as secondary metabolites (natural products), which are crucial determinants in fungal development and metabolism and actively shape interactions with other organisms (Keller, 2018). Some secondary metabolites produced by most pathogenic fungi cause harm and damage host plants. MMC is different from other reaction reagent. In our study, this compound is a novel phytotoxin compound isolated from *R. solani* AG-3 TB which decreases the chlorophyll content and damages the cellular structure. According to the content of AOS (H_2_O_2_ and O^2−^) in leaves, the defense system of plants may be stimulated by MMC. This is the first report to reveal that MMC is a phytotoxin that causes the destruction of leaves’ tissues. However, the toxicological mechanism, synthetic pathway, and related pathogenic genes of this toxic substance are still unclear. Therefore, we should pay more attention to key genes in the synthetic pathway of MMC to reveal the toxic mechanism for *R. solani* in subsequent studies. Collectively, our results provided valuable insights into the toxin compound in *R. solani*, and the potential pathogenic mechanism of the toxin compound in plants was validated.

## Data availability statement

The original contributions presented in the study are included in the article/**Supplementary Material**. Further inquiries can be directed to the corresponding authors.

## Author contributions

XL: Data curation, Formal Analysis, Methodology, Resources, Software, Writing – original draft, Writing – review & editing. MA: Resources, Writing – original draft, Writing – review & editing. HH: Software, Writing – original draft. BL: Formal Analysis, Writing – original draft. SG: Writing – original draft. YX: Writing – original draft. LJ: Resources, Writing – original draft. CX: Methodology, Writing – original draft. CZ: Project administration, Writing – original draft. YW: Funding acquisition, Project administration, Writing – review & editing.
