# Supplementary material for: Identification and physiological activity of (methoxymethyl)triphenylphosphonium chloride as a new phytotoxin isolated from Rhizoctonia solani AG-3 TB
Source: Front Plant Sci. 2023 Nov 17;14:1264567. doi: 10.3389/fpls.2023.1264567 (PMC10693298; doi:10.3389/fpls.2023.1264567)
Supplement: Supplementary file 1 [file Table_1.docx]

**Supplementary Materials**

**Table S1.** The elemental identification of toxin compound

| Repeat | Percentage content (％) | | | |
| --- | --- | --- | --- | --- |
|  | C | H | O | N |
| 1 | 69.84 | 5.72 | 4.59 | 0 |
| 2 | 69.87 | 5.79 | 4.56 | 0 |
| Average value | 69.86 | 5.76 | 4.58 | 0 |

**Table S2.** The hydrogen spectroscopy of toxin compound

| Chemical shift (ppm) | Proton number | Peak shape | Proton chemical shift (ppm) | Ascription | Remarks |
| --- | --- | --- | --- | --- | --- |
| 7.81 | 3H | m | 7.26 | 3H_6_ | 2JPH=4.8Hz |
| 7.65 | 6H | m | 7.62 | 6H_4_ |  |
| 7.62 | 6H | m | 7.81, 7.65 | 6H_5_ |  |
| 5.24 | 2H | s | / | H_7_ |  |
| 3.49 | 3H | s | / | H_1_ |  |

**Table S3.** The carbon spectroscopy of toxin compound

| Chemical shift (ppm) | Ascription | Proton chemical shift（ppm） | Long-range proton chemical shift (ppm) |
| --- | --- | --- | --- |
| 135.5 (d, 4JCP=3.1Hz) | 3C_6_ | 7.81 | 7.65 |
| 133.8 (d, 2JCP=10.2Hz) | 6C_4_ | 7.65 | 7.81 |
| 130.1 (d, 3JCP=12.6Hz) | 6C_5_ | 7.62 | / |
| 115.9 (d, 1JCP=86.1Hz) | 3C_3_ | / | 7.62, 5.24 |
| 65.4 (d, 1JCP=89.8Hz) | C_2_ | 5.24 | 3.49 |
| 62.0 (d, 3JCP=12.5Hz) | C_1_ | 3.49 | 5.24 |
